# Modification of the FoxP3 Transcription Factor Principally Affects Inducible T Regulatory Cells in a Model of Experimental Autoimmune Encephalomyelitis

**DOI:** 10.1371/journal.pone.0061334

**Published:** 2013-04-08

**Authors:** Johan Verhagen, Bronwen R. Burton, Graham J. Britton, Ella R. Shepard, Stephen M. Anderton, David C. Wraith

**Affiliations:** 1 School of Cellular and Molecular Medicine, University of Bristol, Bristol, United Kingdom; 2 Centre for Multiple Sclerosis Research and Centre for Immunology, Infection and Evolution, Queen's Medical Research Institute, Medical Research Council/University of Edinburgh Centre for Inflammation Research, Edinburgh, United Kingdom; University of Florence, Italy

## Abstract

T regulatory (Treg) cells expressing the transcription factor FoxP3 play a key role in protection against autoimmune disease. GFP-FoxP3 reporter mice have been used widely to study the induction, function and stability of both thymically- and peripherally-induced Treg cells. The N-terminal modification of FoxP3, however, affects its interaction with transcriptional co-factors; this can alter Treg cell development and function in certain self-antigen specific animal models. Interestingly, Treg cell function can be negatively or positively affected, depending on the nature of the model. In this study, we focused on the effect of the GFP-FoxP3 reporter on Treg cell development and function in the Tg4 mouse model. In this model, T cells express a transgenic T cell receptor (TCR) specific for the Myelin Basic Protein (MBP) peptide Ac1-9, making the animals susceptible to experimental autoimmune encephalomyelitis (EAE), a disease akin to multiple sclerosis in humans. Unlike diabetes-susceptible mice, Tg4 FoxP3^gfp^ mice did not develop spontaneous autoimmune disease and did not demonstrate augmented susceptibility to induced disease. Concurrently, thymic generation of natural Treg cells was not negatively affected. The induction of FoxP3 expression in naive peripheral T cells was, however, significantly impaired as a result of the transgene. This study shows that the requirements for the interaction of FoxP3 with co-factors, which governs its regulatory ability, differ not only between natural and inducible Treg cells but also between animal models of diseases such as diabetes and EAE.

## Introduction

T regulatory (Treg) cells expressing the transcription factor FoxP3 play an indispensible role in the maintenance of peripheral homeostasis and avoidance of autoimmune disease. Loss or reduced function of FoxP3 leads to severe immunological disorders characterized by lymphocyte hyperproliferation, organ infiltration and autoimmune disease, both in man and mouse [1], [2]. The FoxP3^tm2Ayr^ reporter allele [3], which encodes FoxP3 fused with enhanced Green Fluorescence Protein (eGFP) at its N-terminus, has been used widely to study the role of FoxP3 in a broad range of settings. Two recent reports, however, suggest that this modification of FoxP3 may alter its function in models of autoimmune diabetes or arthritis [4], [5]. Interestingly, both groups found that diabetes onset and severity were exacerbated in NOD mice carrying the GFP transgene, whereas Darce *et al* found disease to be ameliorated in the K/BxN arthritis model. Although FoxP3 is generally seen as the master regulator of Treg cell function, it needs to interact with a host of co-factors (as many as 361) to exert its regulatory effect [6], [7]. In the GFP-FoxP3 fusion protein, the interaction of some of these factors with the N-terminus of FoxP3 is abolished, whereas other interactions are augmented [4], [8]. This differential effect may account for the distinct outcomes in the different disease models. For example, altered IRF4 function may explain the difference between the T helper 1 (Th1) cell-mediated disease in the diabetes model and the Th2-dependent arthritis model [5].

In order to better understand the distinct effects of the N-terminal modification of FoxP3 in different disease models, we crossed Tg4 mice [9], which carry a transgenic T cell receptor (TCR) specific for the myelin basic protein (MBP) peptide Ac1-9 (Ac-ASQKRPSQR), with FoxP3^gfp^ (FoxP3^tm2Ayr^) C57BL/6 mice to create Tg4 FoxP3^gfp^ mice. Tg4 mice are susceptible to Th1-initiated development of Experimental Autoimmune Encephalomyelitis EAE), a model for multiple sclerosis [10]. Spontaneous autoimmune disease in Tg4 mice is controlled by functional FoxP3^+^ Treg cells, as Rag-deficient Tg4 mice, which are naturally devoid of Treg cells, develop encephalomyelitis at 11–12 weeks of age [11]. In this study, we looked at both natural Treg (nTreg) cells generated in the thymus upon recognition of self antigen and inducible Treg (iTreg cells), which are generated from naive conventional T (Tconv) cells in the periphery in the presence of Transforming Growth Factor-β (TGF-β) (reviewed by Josefowicz *et al*., [6]). Tg4 FoxP3^gfp^ mice did not demonstrate an augmented susceptibility to autoimmune disease nor did they demonstrate altered thymic generation of nTreg cells. In vitro differentiation of iTreg cells was, however, impaired in cells expressing the GFP-FoxP3 allele, particularly under conditions suboptimal for induction.

## Materials and Methods

### Ethics statement

All experiments were carried out under a UK Home Office Project License and were subject to assessment by the University of Bristol ethical review committee.

### Mice

C57BL/6 FoxP3^tm2Ayr^ mice, created by the group of A.Y. Rudensky [3], were crossed with CD45.2^+^ Tg4 mice [9] and backcrossed 4 times to obtain the Tg4 FoxP3^gfp^ strain. These, as well as Tg4 CD45.1^+^, H2URagKO and B10.PL mice, were bred under specific pathogen free conditions.

### Peptides

The acetylated N-terminal peptide of murine MBP, Ac1-9 (Ac-ASQKRPSQR) was custom synthesized (purity>85%; GL Biochem (Shanghai) Ltd.).

### T cell isolation

CD4^+^CD62L^+^ naive T cells were isolated magnetically from splenocytes using a naive T cell isolation kit (Miltenyi Biotec) according to the manufacturer's recommendations.

### Flow cytometry

Flow cytometric analysis was performed using an LSR II flow cytometer (BD). Cell phenotypes were analyzed using combinations of anti-GITR-PE, anti-PD-1-PE, anti-FoxP3-PE or -efluor450, anti-CD25-PE or- PECy7, anti-CD45.2-PerCPCy5.5, anti-Helios-AlexaFluor647, anti-CD103-APC, anti-CD8-APC, anti-CD4-AlexaFluor700 (all from eBioscience), anti-CD45.1-APC (BD bioscience) and CD103-PerCPCy5.5 (Biolegend) antibodies. Fixable viability dye eFluor780 (eBioscience) was used in all experiments to exclude dead cells. Results were analyzed using FlowJo analysis software (Tree Star, Inc.).

### iTreg cell generation

CD4^+^CD62L^+^ naive splenic T cells were cultured in vitro for 7 days in the presence of 100 U/ml rhIL-2 (R&D systems) and 10 ng/ml rhTGF-β_1_ (Peprotech). Cells were stimulated either with anti-CD3e (1 µg/ml) and anti-CD28 (2 µg/ml) plate-bound antibody (eBioscience) or MBP Ac1-9 peptide at 0.01 or 0.1 µg/ml, in the presence of irradiated B10.PL splenocytes as antigen-presenting cells. The level of FoxP3 induction was assessed by flow cytometry prior to in vitro co-culture or transfer in vivo.

### Proliferation/suppression assays

For in vitro experiments, both iTreg cells (CD45.2^+^) and naive CD4^+^CD62L^+^ CD45.1^+^ T cells were labeled with 5 µM Cell Proliferation Dye (CPD) eFluor450 (eBioscience). 5×10^5^ naive T cells, together with the indicated ratio of iTreg cells, were stimulated for 72 h with 10 µg/ml MBP Ac1-9 peptide in the presence of 1×10^6^ irradiated B10.PL splenocytes serving as antigen-presenting cells, in 48-well plates. For in vivo experiments, CPD eFluor450 labeled CD45.1^+^ naive cells (males, 2×10^6^, females 5×10^6^) and CD45.2^+^ iTreg cells were transferred intraperitoneally to H2URagKO recipients. Cells were recovered from the spleen on day 5 and proliferation as indicated by CPD eFluor450 dilution was assessed by flow cytometry.

### Induction and scoring of EAE

14–15 week-old mice were primed subcutaneously at the base of the tail with 200 µg of MBP Ac1-9 in 0.1 ml of a sonicated emulsion consisting of an equal volume of complete Freund's adjuvant (CFA) and PBS containing 4 mg/ml of heat-killed Mycobacterium Tuberculosis (both from Difco). On days 0 and 2, 200 ng of Pertussis toxin (Sigma Aldrich) was administered intraperitoneally in 0.5 ml of PBS. EAE was assessed twice daily for 21 days with the following scoring system: 0, no signs; 1, flaccid tail; 2; impaired righting reflex and/or gait; 3, hind limb paralysis; 4, forelimb and hind limb paralysis; 5, moribund.

### Statistical analysis

Data was analyzed for statistical significance using GraphPad Prism software.

## Results

### Modification of FoxP3 does not significantly alter the number of nTreg cells

**Figure 1:**
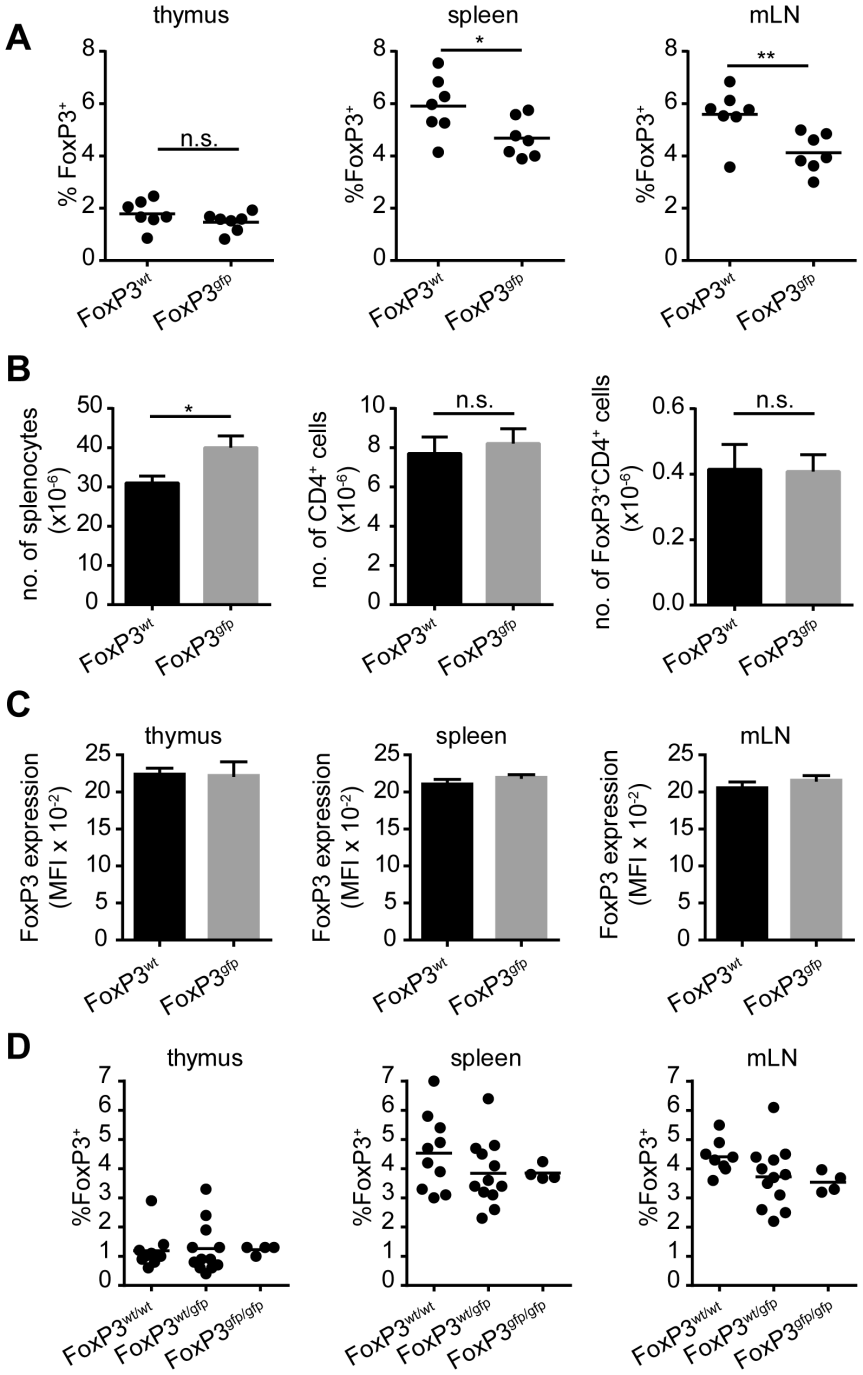
Young Tg4 FoxP3^gfp^mice have unaltered levels of FoxP3 expression. A. FoxP3 expression by CD4^+^CD8^−^ thymocytes or CD4^+^ cells from the spleen and mesenteric lymph nodes of male Tg4 FoxP3^wt^ and Tg4 FoxP3^gfp^ mice aged 5–7 weeks, directly ex vivo. Horizontal bar indicates mean. * p = 0.0329, ** p = 0.0084, n.s. = not significant. 2-tailed, unpaired student's t test, n = 7 each. B. Total number of splenocytes, CD4^+^ T cells and FoxP3^+^CD4^+^ Treg cells in the spleen of male Tg4 mice aged 5–7 weeks. Data displayed as mean, error bar indicates SEM. n = 3 each, * p = 0.0108, n.s. = not significant. 2-tailed, unpaired student's t test. C. Mean fluorescence intensity of FoxP3 staining in Treg cells in the thymus, spleen and mLN of 5–7 week old male Tg4 mice. Data displayed as mean, error bar indicates SEM. n = 3 each, no statistical difference, unpaired, 2-tailed student's t test. D. Frequency of FoxP3 expression on CD4^+^CD8^−^ thymocytes or CD4^+^ cells from the spleen or mLN of Tg4 FoxP3^wt/wt^, Tg4 FoxP3^wt/gfp^ or Tg4 FoxP3^gfp/gfp^ females aged 5–8 weeks. FoxP3^wt/wt^ n = 8, FoxP3^wt/gfp^ n = 12, FoxP3^gfp/gfp^ n = 4. No statistically significant differences, Tukey's multiple comparison test.

In order to study the effect of fusing GFP to the FoxP3 protein on the generation of nTreg cells, we looked at the expression of FoxP3 by CD4^+^ T cells in young Tg4 FoxP3^wt^ and FoxP3^gfp^ males (5–7 weeks of age). The frequency of FoxP3 expression by CD4^+^CD8^−^ cells isolated from the thymus was identical regardless of transgene expression (Figure 1A). In the spleen, the frequency of FoxP3 expression by CD4^+^ T cells showed a trend towards lower expression in mice carrying the GFP-FoxP3 transgene (Figure 1A), yet the absolute number of CD4^+^FoxP3^+^ cells in the spleen did not change significantly (Figure 1B). Next, we looked at FoxP3 expression in the mesenteric lymph nodes (mLN) as these are a major site of peripheral FoxP3 induction [6]. Unlike the thymus, the mLN showed a reduction in the frequency of FoxP3 expression in CD4^+^ T cells from Tg4 FoxP3^gfp^ mice, indicating that in the periphery GFP^pos^ Treg cells may demonstrate impaired proliferation, survival or induction (figure 1A). The average expression of FoxP3 per cell (as determined by the Mean Fluorescence Index (MFI) of anti-FoxP3 staining) in male Tg4 FoxP3^wt^ and Tg4 FoxP3^gfp^ animals did not vary as a result of the transgene, in any of the organs examined (Figure 1C). The *foxp3* gene is located on the X chromosome; so, due to the process of X chromosome inactivation, approximately half of Treg cells are expected to express wild-type and half transgenic FoxP3 in heterozygous females. Although the frequency of FoxP3 expression in the thymus, spleen and mLN of Tg4 FoxP3^wt/wt^, FoxP3^wt/gfp^ and FoxP3^gfp/gfp^ females (aged 5–8 weeks) did not differ with statistical significance, a trend towards lower FoxP3 frequency in the mLN was observed depending on the level of transgene expression (Figure 1D).

### GFP^pos^ cells inTg4 FoxP3^wt/gfp^ females demonstrate greater expression of Treg cell functional antigens

**Figure 2:**
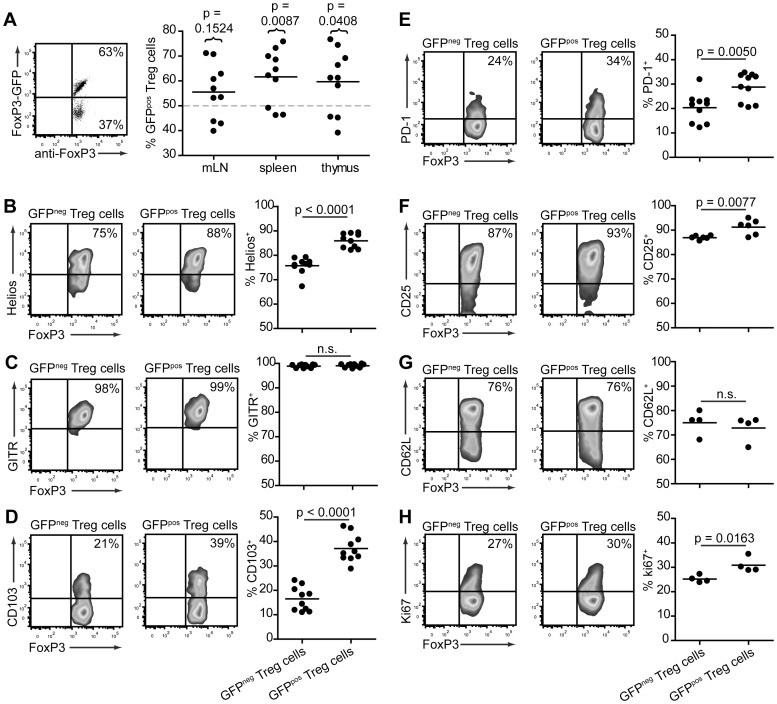
GFP^pos^ Treg cells display a potentially more suppressive phenotype in Tg4 FoxP3^wt/gfp^ females. A. Representative dot plot (splenocytes, left) and distribution graph (right) showing the frequency of GFP expression in CD4^+^FoxP3^+^ cells from the thymus, spleen and mLN of Tg4 FoxP3^wt/gfp^ females aged 5–8 weeks. Horizontal dotted line represents the predicted frequency. P values show significance of actual vs predicted value. 2-tailed, paired student's t test. n = 10 each. B-H. Representative, smoothed, cytometry plots and distribution graphs showing the frequency of marker expression on GFP^pos^ and GFP^neg^ splenic CD4^+^FoxP3^+^ Treg cells in Tg4 FoxP3^wt/gfp^ females aged 5–10 weeks. P values represent significance determined by 2-tailed, unpaired student's t test. n.s. = not significant. B. Helios, n = 10. C. GITR, n = 10. D. CD103, n = 10. E. PD-1, n = 10. F. CD25, n = 6. G. CD62L, n = 4. H. Ki67, n = 4.

X chromosome inactivation is generally considered to be a random process. Therefore, in Tg4 FoxP3-GFP^wt/gfp^ females, 50% of all CD4^+^FoxP3^+^ Treg cells were expected to be GFP^pos^ and 50% GFP^neg^. This, however, turned out not to be the case. As shown in Figure 2A, the majority of animals (aged 5–8 weeks) investigated demonstrated a higher than anticipated frequency of GFP^pos^ Treg cells in the thymus, spleen and mLN. Interestingly, this result is the opposite of that reported in K/BxN, C57BL/6 and NOD mice, where the frequency of GFP^pos^ Treg cells in FoxP3^wt/gfp^ females was generally lower than 50% [5].

In the next step, we examined the expression of antigens commonly associated with Treg cell function and Treg cell subtypes to try and establish whether both GFP^neg^ and GFP^pos^ Treg cells in Tg4 FoxP3^wt/gfp^ females are genuine and functional suppressor cells. Helios is an Ikaros-family transcription factor predominantly expressed in FoxP3^+^ Treg cells. It has been suggested that Helios can be used to distinguish nTreg cells from iTreg cells; however, this theory has been challenged, including in the Tg4 model [12], [13], [14]. Here, we found a greater percentage of Helios expressing cells among GFP^pos^ Treg cells than GFP^neg^ Treg cells in Tg4 FoxP3^wt/gfp^ females (Figure 2B). The glucocorticoid-induced TNF receptor (GITR) has been suggested to be a more accurate marker for Treg cells than CD25 as it is also expressed on CD25^−^FoxP3^+^ cells with regulatory function [15]. It was found to be expressed on all FoxP3^+^ Treg cells in Tg4 FoxP3^wt/gfp^ mice, regardless of GFP expression (Figure 2C). CD103, an α/β integrin that may identify a more potent subpopulation of Treg cells [16], showed a higher frequency of expression on GFP^pos^ than GFP^neg^ Treg cells in our model (Figure 2D). Similarly, the negative co-stimulatory molecule Programmed Death-1 (PD-1), which affects both the generation and function of Treg cells [17], was also more commonly expressed on GFP^pos^ than GFP^neg^ Treg cells, although the difference was less striking than found for CD103 or Helios (Figure 2E). The classic Treg cell identifier CD25 was expressed highly on both GFP^neg^ and GFP^pos^ FoxP3^+^ Treg cells, with a small increase in the latter (Figure 2F). Considering that most of these Treg cell markers are also indicators of T cell activation, we examined the expression of CD62L, a selectin commonly associated with naive T cells, and Ki67, a nuclear protein present in dividing but not resting T cells, to establish whether there might be a difference in the activation status of GFP^neg^ and GFP^pos^ Treg cells in Tg4 FoxP3^wt/gfp^ females. The expression of CD62L was not significantly different between GFP^neg^ and GFP^pos^ Treg cells (Figure 2G). Ki67 expression was slightly elevated in GFP^pos^ Treg cells (Figure 2H), but mean expression was much higher in both GFP^neg^ Treg cells (25.18±0.74%) and GFP^pos^ Treg cells (30.85±1.55%) than in CD4^+^ Tconv cells of the same mice (7.11±0.34%, data not shown). The slight increase in Ki67 expression but similar CD62L expression may suggest augmented homeostatic turnover of GFP^pos^ Treg cells, yet this is not reflected in the frequency of GFP^pos^ Treg cells in the periphery versus the thymus (Figure 2A). In conclusion, the phenotype of GFP^pos^ Treg cells in Tg4 FoxP3^wt/gfp^ females, suggests they may be more potent immune regulators than their wild-type counterparts.

### Aged Tg4 FoxP3^gfp^ mice harbor lower peripheral Treg cell numbers but do not develop spontaneous autoimmune disease

**Figure 3:**
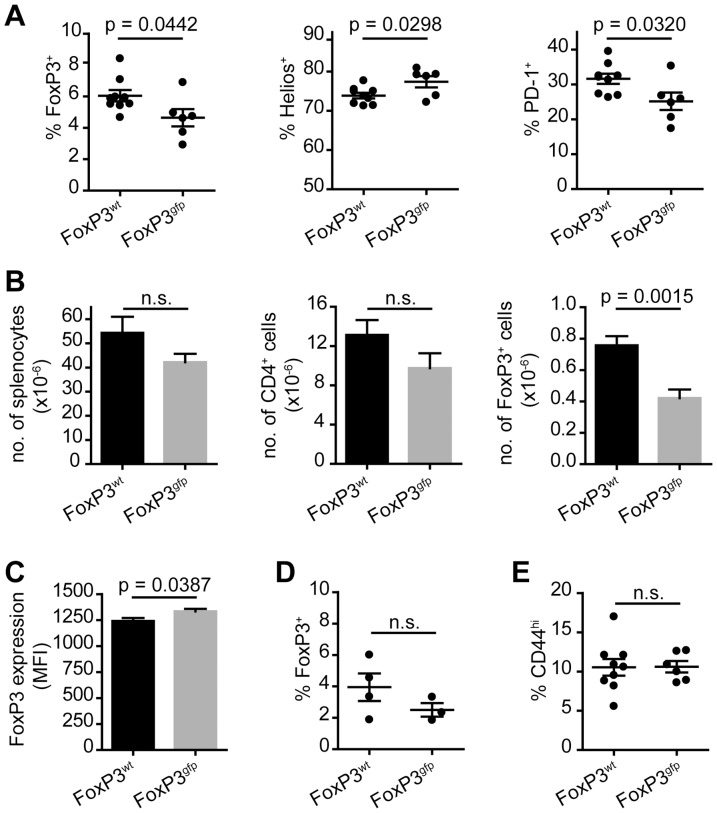
Lower number of peripheral FoxP3^+^ Treg cells in aged Tg4 FoxP3^gfp^ males. A. Frequency of FoxP3 expressing CD4^+^ T cells in the spleen of Tg4 FoxP3^wt^ and Tg4 FoxP3^gfp^ males aged 16–24 weeks (left) and the frequency of Helios and PD-1 expression gated on CD4^+^FoxP3^+^ splenocytes (middle and right). FoxP3^wt^ n = 9, FoxP3^gfp^ n = 6. Data represented as individual scores, with mean±SEM. B. Total number of splenocytes, CD4^+^ T cells or CD4^+^FoxP3^+^ Treg cells in the spleen. FoxP3^wt^ n = 9, FoxP3^gfp^ n = 6. Data represented as mean + SEM. C. Mean fluorescence intensity of FoxP3 staining in Treg cells in the spleen. FoxP3^wt^, n = 9, FoxP3^gfp^ n = 6. Data represented as mean+SEM. D. Frequency of Foxp3 expression by CD4^+^ cells in the mLN. FoxP3^wt^ n = 4, FoxP3^gfp^ n = 3. Data represented as individual scores, with mean±SEM. E. Percentage of CD44^hi^ CD4^+^ T cells in the spleen. FoxP3^wt^ n = 9, FoxP3^gfp^ n = 6. Data represented as individual scores, with mean±SEM. P values represent significance determined by 2-tailed, unpaired student's t test. n.s. = not significant.

Tg4 mice are susceptible to induced EAE but do not develop disease spontaneously. To determine whether the modification of FoxP3 would increase susceptibility to spontaneous autoimmune disease, despite the lack of obvious detrimental alterations to their Treg cells as described above, we followed groups of Tg4 FoxP3^wt^ and Tg4 FoxP3^gfp^ males until 16–24 weeks of age. Tg4 Rag^−/−^ mice, which are naturally devoid of Treg cells, develop spontaneous CNS autoimmune disease at 11–12 weeks of age [11]. Tg4 FoxP3^gfp^ mice, however, did not demonstrate any sign of disease even at 24 weeks of age (data not shown), thus indicating that immune regulation in these animals was not severely impaired. However, in contrast to younger mice, both the frequency and the absolute number of FoxP3^+^ Treg cells in the spleen of aged males were significantly lower in Tg4 FoxP3^gfp^ compared to Tg4 FoxP3^wt^ individuals (Figure 3A and 3B). This seemed to have resulted from a greater expansion or peripheral induction of FoxP3^+^ Treg cells in the Tg4 FoxP3^wt^ mice, rather than a loss of expression in the Tg4 FoxP3^gfp^ mice, as the latter had the same number of FoxP3^+^ cells in the spleen at 16–24 weeks as at age 5–7 weeks (Figures 1B and 3B). Although differences were small, Treg cells in older Tg4 FoxP3^gfp^ males demonstrated elevated levels of Helios, but lower levels of PD-1 expression than in Tg4 FoxP3^wt^ mice (Figure 3A). The mean level of FoxP3 expression per Treg cell (as determined by MFI) was only moderately lower in Tg4 FoxP3^wt^ than Tg4 FoxP3^gfp^ animals at age 16–24 weeks (Figure 3C). In the mLN, a similar difference in the frequency of FoxP3 expression as in the spleen was found between Tg4 FoxP3^wt^ and Tg4 FoxP3^gfp^ mice (Figure 3D). Finally, the frequency of CD44^hi^ memory CD4^+^ Tconv cells was low and comparable between the two groups, supporting the notion that despite the lack of peripheral Treg cell accumulation, Tg4 FoxP3^gfp^ mice were not more susceptible to spontaneous activation of self-reactive T cells and subsequent autoimmune disease (Figure 3E).

### In vitro generation of iTreg cells is impaired in Tg4 FoxP3^gfp^ naive CD4^+^ cells

**Figure 4:**
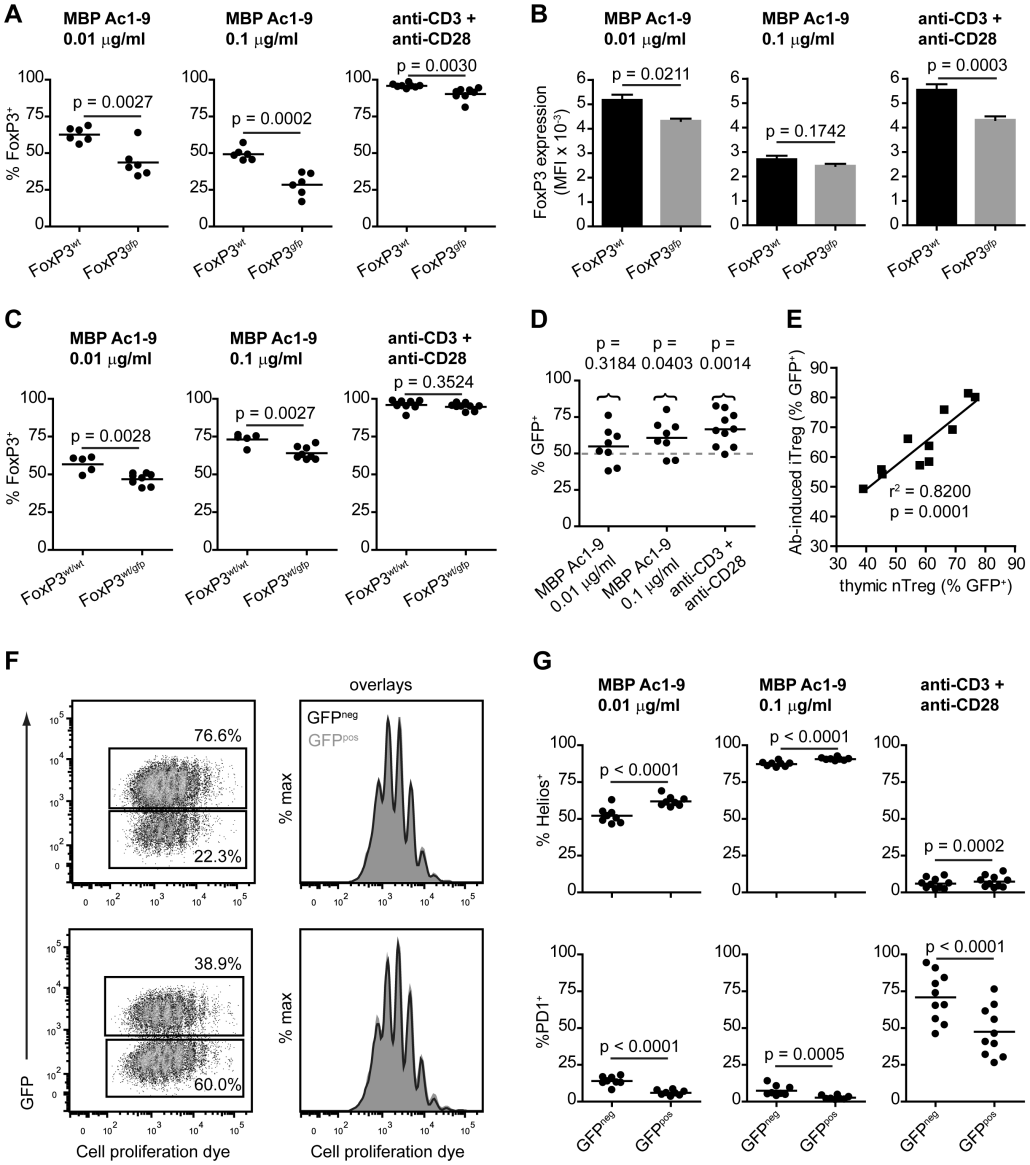
Impaired FoxP3 induction in naive Tg4 FoxP3^gfp^ CD4^+^ T cells. A. In vitro FoxP3 induction in male, naive CD4^+^CD62L^+^ T cells by stimulation with peptide and antigen-presenting cells or plate-bound anti-CD3 and anti-CD28 antibody in the presence of IL-2 and TGF-β1 for 7 days. n = 6 each for peptide stimulation, n = 8 each for antibody stimulation. B. Mean fluorescence intensity of anti-FoxP3 antibody staining in FoxP3^+^ iTreg cells. Data represented as mean + SEM. n = 6 each for peptide stimulation, n = 8 each for antibody stimulation. C. In vitro FoxP3 induction in female Tg4 FoxP3^wt/wt^ and Tg4 FoxP3^wt/gfp^ naive CD4^+^ T cells. Peptide stimulation; FoxP3^wt/wt^ n = 5, FoxP3^wt/gfp^ n = 8. Antibody stimulation; FoxP3^wt/wt^ n = 10, FoxP3^wt/gfp^ n = 9. D. Frequency of GFP expression in FoxP3^+^CD4^+^ iTreg cells generated from Tg4 FoxP3^wt/gfp^ naive T cells using peptide or plate-bound antibody. Peptide stimulation; n = 8, antibody stimulation; n = 10. E. Correlation plot of the frequency of GFP expression in antibody-induced FoxP3^+^ iTreg cells and thymic FoxP3^+^ nTreg cells from the same female Tg4 FoxP3^wt/gfp^ mice. n = 11. F. Proliferation of CD4^+^ T cells during antibody-mediated iTreg cell generation. Naive female CD4^+^CD62L^+^ Tg4 FoxP3^wt/gfp^ cells were labeled with 5 µM CPD-eFluor450 prior to 7-day culture with IL-2 and TGF-β_1_. Plots gated on FoxP3^+^ Treg cells. Shown are proliferation of GFP^neg^ and GFP^pos^ Treg cells from mice with a high (top row) or low (bottom row) frequency of GFP expression. Histogram overlays show proliferation of GFP^neg^ (black, open line) and GFP^pos^ (grey, filled line) Treg cells. 2 out of 4 identical experiments (each in triplicate) shown. G. Frequency of Helios and PD-1 expression in GFP^pos^ and GFP^neg^ FoxP3^+^CD4^+^ iTreg cells generated from splenic, naive CD4^+^ T cells of Tg4 FoxP3^wt/gfp^ mice. n = 8. A, B and C; 2-tailed, unpaired student's t test. D. 2-tailed, paired t test on actual measurements versus predicted value of 50%. F and G; 2-tailed, paired student's t test.

The lower number of peripheral FoxP3^+^ Treg cells in aged Tg4 FoxP3^gfp^ males suggests that either peripheral induction, survival or expansion of Treg cells is negatively affected by the transgene. The finding that a greater fraction of splenic GFP^pos^ than GFP^neg^ Treg cells in Tg4 FoxP3^wt/gfp^ females express the indicator of cell division Ki67 (Figure 2H) suggested peripheral expansion was not impaired as a result of the transgene. Therefore, we studied the in vitro induction of FoxP3 expression in naive CD4^+^CD62L^+^ splenic T cells under the influence of IL-2 and TGF-β_1_. We compared iTreg cell generation using either a combination of plate-bound anti-CD3 and anti-CD28 or cognate MBP Ac1-9 peptide presented by irradiated APC as a stimulus, as previous findings had suggested that the resulting cells may have different characteristics [13]. In males, the percentage of FoxP3 induction was markedly decreased in Tg4 FoxP3^gfp^ CD4^+^ T cells compared to Tg4 FoxP3^wt^ CD4^+^ T cells, either using different concentrations of peptide or antibody stimulation (Figure 4A). Furthermore, unlike what we observed in nTreg cells, Tg4 FoxP3^gfp^ iTreg cells showed a lower level of FoxP3 expression (MFI) than Tg4 FoxP3^wt^ iTreg cells (Figure 4B). A similar effect was observed in Tg4 FoxP3^wt/wt^ versus Tg4 FoxP3^wt/gfp^ females, albeit less distinct, as expected, because only a fraction of Treg cells carried the transgene in heterozygous mice (Figure 4C). Remarkably, although the GFP-FoxP3 transgene clearly impaired iTreg cell generation, the frequency of GFP^pos^ cells among Tg4 FoxP3^wt/gfp^ iTreg cells was still higher on average than the 50% expected in the case of random X chromosome inactivation (Figure 4D). The relative expression of GFP in FoxP3^+^ iTreg cells generated from Tg4 FoxP3^wt/gfp^ naive CD4^+^ splenocytes seemed to correlate with the success of FoxP3 induction in general, as the method that gave the highest level of FoxP3 expression, i.e. antibody stimulation which gave around 95% FoxP3 induction, also gave the highest percentage of GFP^pos^ FoxP3^+^ iTreg cells. In these antibody-generated Tg4 FoxP3^wt/gfp^ iTreg cells in particular, the frequency of GFP^pos^ cells showed a strong correlation with the frequency of GFP^pos^ cells in thymic nTreg cells of the same mouse (Figure 4E). This would indicate that the frequency at which GFP^pos^ iTreg or nTreg cells are generated in Tg4 FoxP3^wt/gfp^ mice depends not only the efficacy of their induction, but is determined largely prior to FoxP3 expression, either in the thymus or periphery. This could, for example, result from non-random X chromosome inactivation, where either the wild-type or transgenic allele is preferentially silenced. To further support the notion that the frequency of GFP^pos^ cells among FoxP3^wt/gfp^ iTreg cells generated using antibody stimulation of naive T cells was predetermined mostly by a genetic factor rather than a result of a functional difference between GFP^neg^ and GFP^pos^ iTreg cells during differentiation, we studied the rate at which female Tg4 FoxP3^wt/gfp^ CD4^+^ T cells divided during the seven-day in vitro culture. Naive T cells were labeled with Cell Proliferation Dye (CPD)-eFluor450 prior to stimulation with plate-bound anti-CD3 and anti-CD28 antibody in the presence of IL-2 and TGF-β1. As shown in Figure 4F, GFP^neg^ and GFP^pos^ iTreg cells proliferated at the same rate during differentiation. This was confirmed in TG4 FoxP3^wt/gfp^ females with both a high (top row) or low (bottom row) frequency of GFP^pos^ Treg cells.

Finally, we assessed phenotypic differences between GFP^neg^ and GFP^pos^ iTreg cells generated from naive Tg4FoxP3^wt/gfp^ CD4^+^ splenocytes, either using cognate peptide or plate-bound antibody. Expression of the Helios transcription factor was much higher in peptide-generated than antibody-generated iTreg cells from Tg4 FoxP3^wt/gfp^ females, as expected from previous results [13], with the frequency of Helios expression dependent on the concentration of peptide used (Figure 4H, top row). GFP^pos^ iTreg cells showed a moderately higher frequency of Helios expression than GFP^neg^ iTreg cells regardless of the induction method. PD-1 expression showed a pattern opposite to that of Helios expression, with the highest frequency found in antibody-generated iTreg cells and a higher percentages in GFP^neg^ than GFP^pos^ iTreg cells (Figure 4H, bottom row). These findings suggest that the lower number of FoxP3^+^ cells found in the spleen of aged Tg4 FoxP3^gfp^ males reflects an impaired peripheral generation of FoxP3^+^ iTreg cells as a result of the transgene. The GFP-FoxP3 fusion seemed particularly detrimental to FoxP3 induction under conditions of suboptimal iTreg cell generation with peptide, which may be the more physiologically relevant method.

### Tg4 FoxP3^gfp^ and Tg4 FoxP3^wt^ iTreg cells are unstable, in vitro and in vivo

**Figure 5:**
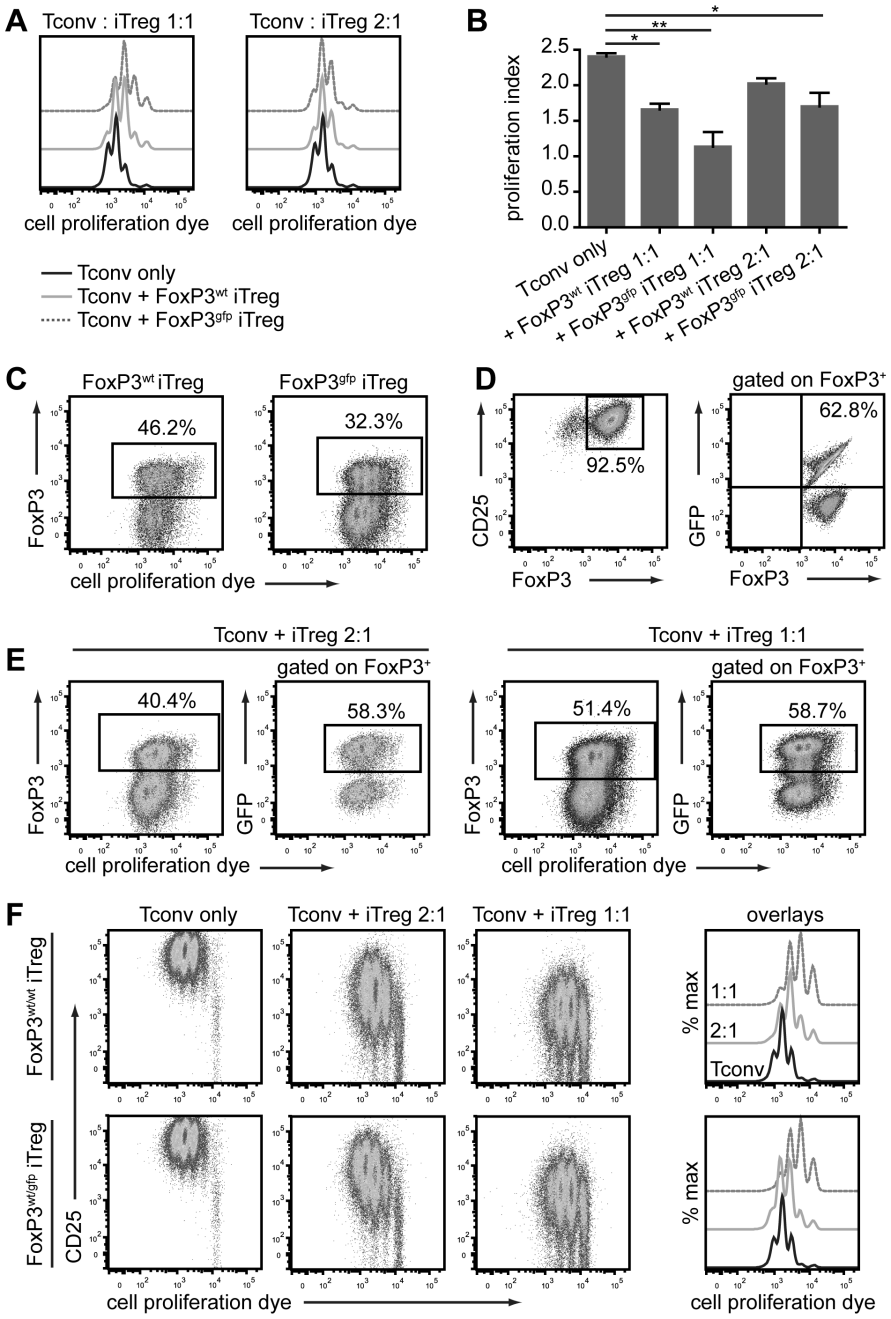
Tg4 FoxP3^gfp^ iTreg cells are suppressive but unstable in vitro. A. Representative histograms of cell proliferation dye dilution in labeled CD45.1^+^ naive CD4^+^ T cells cultured for 3 days with/without Tg4 FoxP3^wt^ or Tg4 FoxP3^gfp^ iTreg cells at a 1∶1 or 2∶1 ratio and stimulated with 10 µg/ml MBP Ac1-9. One representative of three identical experiments in triplicate. B. Proliferation index of naive CD4^+^ cells after 72 h co-culture with/without iTreg cells. Means of 3 identical experiments carried out in triplicate. * p < 0.05, ** p < 0.001, Tukey's multiple comparison test. C. Frequency of FoxP3 expression and proliferation of CD45.2^+^ iTreg cells after 3-day co-culture with naive CD45.1^+^ CD4^+^ T cells. One experiment representative of three carried out in triplicate. Gated on live CD45.2^+^ CD4^+^ T cells. D. Purity of FoxP3^+^ CD4^+^CD25^+^ Tg4 FoxP3^wt/gfp^ iTreg cells and frequency of GFP expression therein, prior to in vitro co-culture with naive T cells. E. Proliferation and FoxP3 retention of CD45.2^+^ Tg4 FoxP3^wt/gfp^ iTreg cells as well as frequency of GFP expression among CD45.2^+^ FoxP3^+^ cells after 3-day co-culture with naive CD45.1^+^ CD4^+^ T cells. F. CD25 expression and cell proliferation dye dilution in naive CD45.1^+^ CD4^+^ T cells after 72-h in vitro co-culture with CD45.2^+^ Tg4 FoxP3^wt/wt^ or Tg4 FoxP3^wt/gfp^ iTreg cells at different ratios. Proliferation dye dilution displayed as dot plot versus CD25 expression, or off-set histogram overlays. D-F. One experiment representative of 3 identical experiments.

To examine whether not only the degree of FoxP3 induction and expression of Treg-associated antigens, but also the functionality of FoxP3-GFP^pos^ iTreg cells was altered, we tested their stability and suppressive ability both in vitro and in vivo. Although iTreg cells are commonly seen as a prime therapeutic target for the induction of immune tolerance, the stability of their phenotype, in particular, raises concerns [18]. For these experiments, antibody-induced iTreg cells were used as these could be generated to a very high level of purity (approximately 95% in Tg4 FoxP3^wt^ cells) with only a marginal reduction in FoxP3 expression (2–3%) in Tg4 FoxP3^gfp^ cells. First, we looked at the ability of male CD45.2^+^ Tg4 FoxP3^wt^ and Tg4 FoxP3^gfp^ iTreg cells to suppress the peptide-induced activation of naive CD4^+^CD62L^+^ Tg4 CD45.1^+^ T cells in vitro. Both iTreg cells and naive Tconv cells were labeled with CPD-eFluor450 to monitor expansion. Tg4 FoxP3^wt^ and Tg4 FoxP3^gfp^ iTreg cells both significantly reduced the proliferation of the Tconv cells at high ratios, although Tg4 FoxP3^gfp^ iTreg cells were the more effective suppressors (Figure 5A and 5B). However, the Tg4 FoxP3^gfp^ iTreg cells were also of a less stable phenotype, with around 2/3 of iTreg cells losing Foxp3 expression after 3 days (Figure 5C). We next examined iTreg cells generated from naive Tg4 FoxP3^wt/gfp^ female CD4^+^ T cells in order to compare the stability of FoxP3 expression in GFP^pos^ and GFP^neg^ iTreg cells in the same culture. In this case, the stability of FoxP3^+^ cells depended on the ratio of iTreg cells to naive CD4^+^ T cells in the culture, with the higher ratio giving greater stability (Figure 5D and 5E). The frequency of GFP^pos^ cells among iTreg cells that had retained FoxP3 expression was only fractionally lower compared to that before culture, indicating that the difference in stability of GFP^pos^ and GFP^neg^ iTreg cells is limited in co-culture. iTreg cells from Tg4 FoxP3^wt/gfp^ females were similarly suppressive to those from Tg4 FoxP3^wt/wt^ mice (Figure 5F).

**Figure 6:**
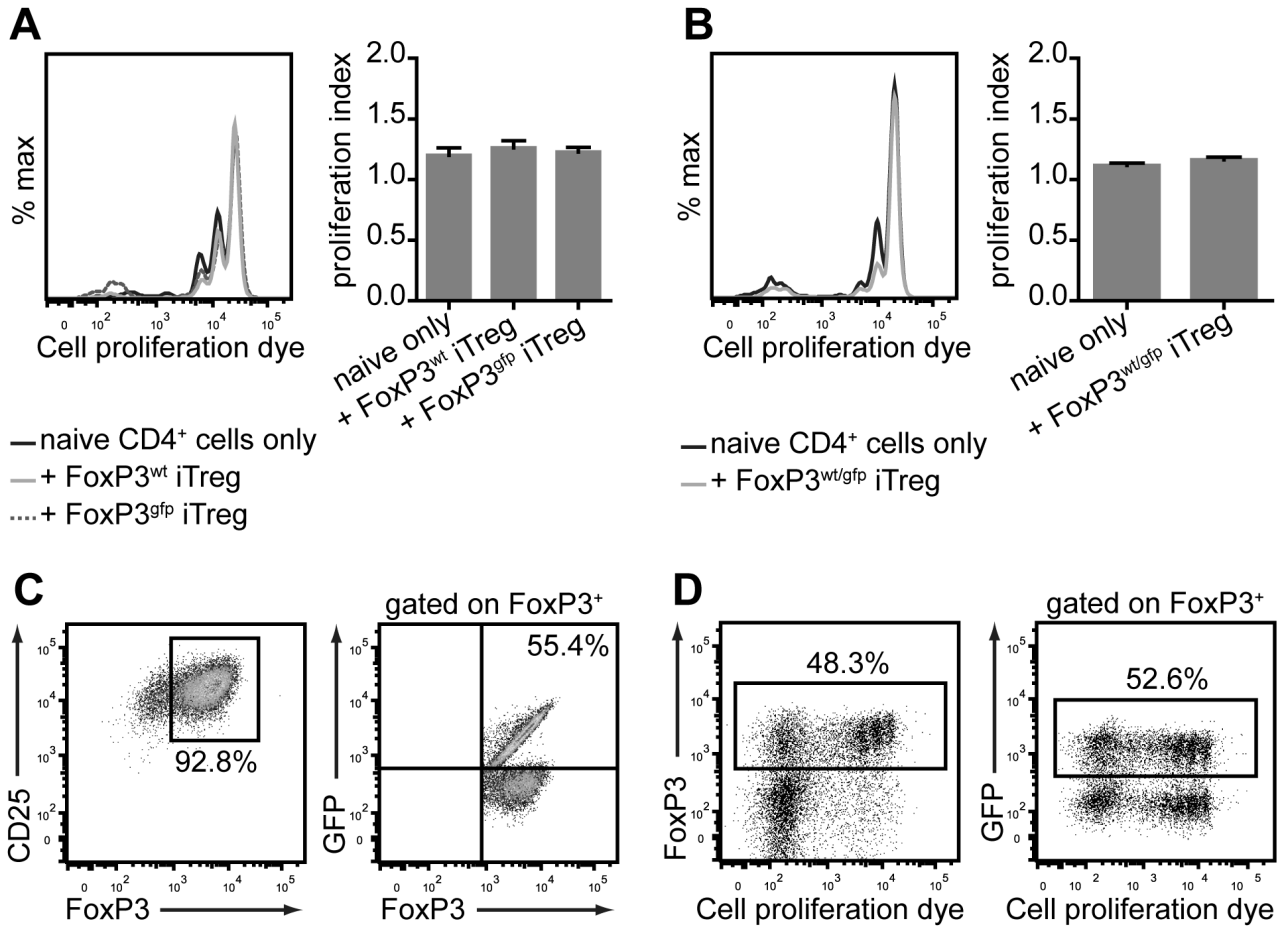
Tg4 iTreg cells are unstable during homeostatic expansion in vivo. A. Representative histogram overlay of cell proliferation dye dilution of labeled male Tg4 CD45.1^+^ CD4^+^ Tconv cells (left) and proliferation index (mean±SEM), 5 days after co-transfer with/without CD45.2^+^ Tg4 FoxP3^wt^ or Tg4 FoxP3^gfp^ iTreg cells into lymphopenic H2URagKO mice. One representative of three identical experiments. B. Representative histogram overlay of cell proliferation dye dilution in labeled female Tg4 CD45.1^+^ CD4^+^ Tconv cells (left) and proliferation index (mean±SEM), 5 days after co-transfer with/without CD45.2^+^ Tg4 FoxP3^wt/gfp^ iTreg cells into lymphopenic H2URagKO mice. One representative of three identical experiments. C. Purity of FoxP3^+^ CD4^+^CD25^+^ Tg4 FoxP3^wt/gfp^ iTreg cells and frequency of GFP expression therein, prior to co-transfer with naive T cells. D. Proliferation and FoxP3 retention of CD45.2^+^ Tg4 FoxP3^wt/gfp^ iTreg cells (left) as well as frequency of GFP expression among CD45.2^+^ FoxP3^+^ cells (right), 5 days after co-transfer with naive CD45.1^+^ CD4^+^ Tconv cells. C and D; One experiment representative of 3 identical experiments.

Considering the unstable phenotype of iTreg cells in these in vitro cultures, it is not unreasonable that the suppressive effect on the proliferation of Tconv cells was, at least in part, due to nutrient depletion in the culture rather than a direct suppressive effect. To address this, we investigated the ability of CD45.2^+^ Tg4 FoxP3^gfp^ iTreg cells to limit homeostatic expansion of CD45.1^+^ CD4^+^CD62L^+^ naive Tconv cells upon transfer to lymphopenic H2URagKO mice. These experiments demonstrated that neither male Tg4 FoxP3^wt^ and Tg4 FoxP3^gfp^iTreg cells nor female Tg4 FoxP3^wt/gfp^ iTreg cells had a significant effect on naive T cell expansion in this setting (Figure 6A and 6B). Interestingly though, Foxp3 expression in Tg4 FoxP3^wt/gfp^ iTreg cells showed a very similar level of stability in vivo as observed in vitro, with approximately half the cells having lost FoxP3 expression 5 days post transfer (Figure 6C and 6D). Furthermore, the frequency of GFP^pos^ cells among iTreg cells that had retained FoxP3 expression after transfer was only moderately reduced, confirming that the phenotype of GFP^pos^ iTreg cells was not much less stable than that of GFP^neg^ iTreg cells when transferred together (Figure 6D).

### Tg4 FoxP3^gfp^ mice do not show augmented susceptibility to induced CNS autoimmune disease

**Figure 7:**
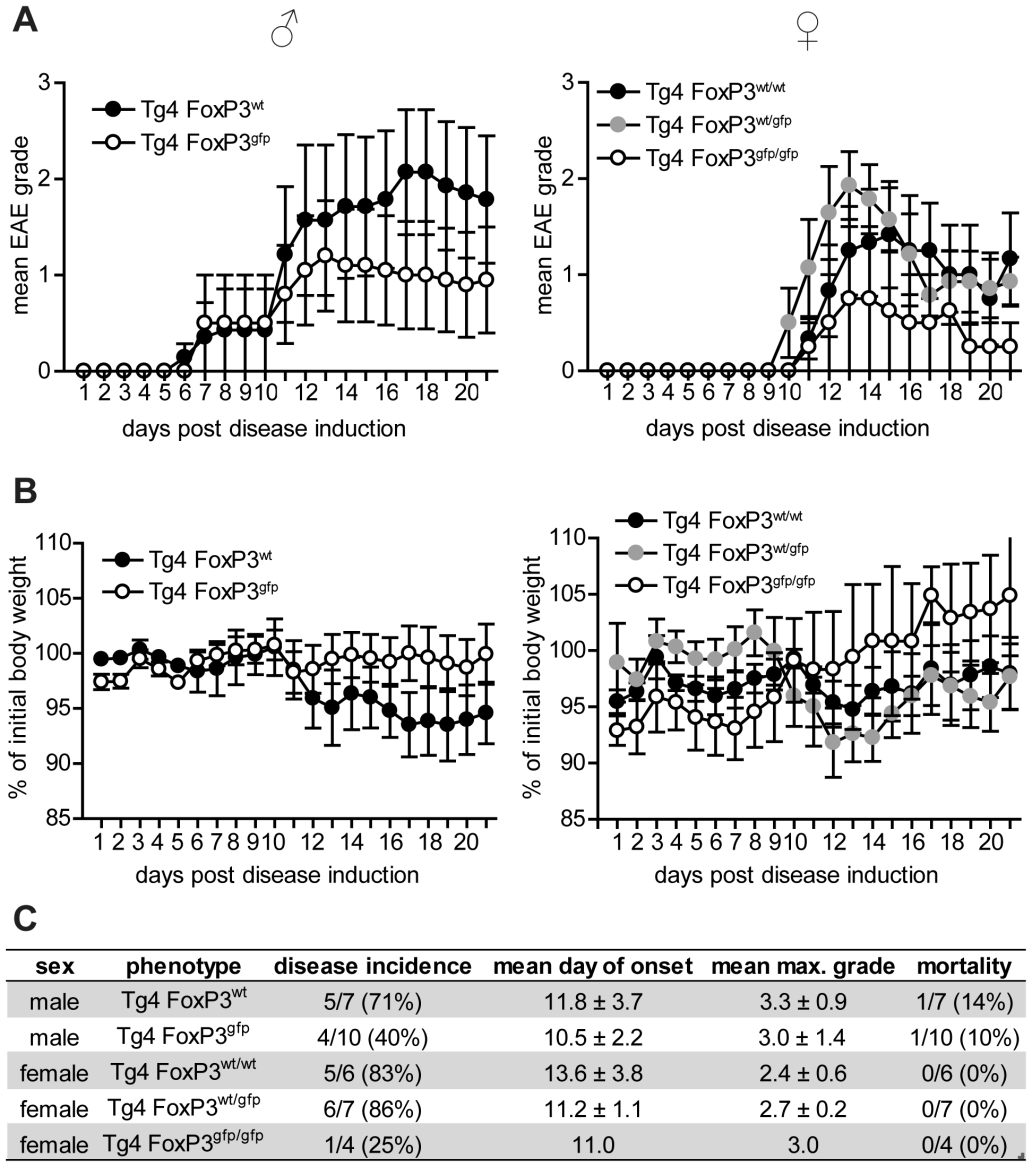
Tg4 FoxP3gfp mice are not more susceptible to induced disease. A. Mean EAE disease scores (± SEM) in male and female Tg4 FoxP3 mice, after induction of disease with MBP Ac1-9 in CFA. Male Tg4 FoxP3^wt^ n = 7, male Tg4 FoxP3^gfp^ n = 10, female Tg4 FoxP3^wt/wt^ n = 6, female Tg4 FoxP3^wt/gfp^ n = 7, female Tg4 FoxP3^gfp/gfp^ n = 4. B. Mean percentage of initial body weight (on day -1)±SEM, after disease induction. C. disease incidence, mean day of onset (± SD), mean maximum disease grade (± SD) and mortality during the first 21 days following disease induction.

Although Tg4 FoxP3^gfp^ mice do not show an increase in the incidence of spontaneous autoimmune disease, the impaired generation of iTreg cells that results from the modification of FoxP3 raised the question whether they display augmented susceptibility to induced EAE. To address this, we immunized both male and female mice (average age of 14-15 weeks) with MBP Ac1-9 peptide in complete Freund's adjuvant (Figure 7 A-C). It was clear from these experiments that neither Tg4 FoxP3^gfp^ males nor Tg4 FoxP3^gfp/gfp^ females demonstrated exacerbated disease compared to wild-type controls. In fact, the disease incidence in both males and females suggested that the GFP-FoxP3 transgene may have provided enhanced protection, although the difference was not as profound as previously reported for spontaneous disease in the K/BxN arthritis model [5].

Overall, we conclude that the modification of FoxP3 in this model selectively affects the peripheral induction of FoxP3 expression, with thymic generation of MBP-specific nTreg cells seemingly unimpaired. The reduced peripheral induction of FoxP3^+^ Treg cells does not hinder the protection against spontaneous or provoked encephalomyelitis in the Tg4 mouse, suggesting that thymic nTreg cells provide an important barrier to the development of this autoimmune disease.

## Discussion

Treg cells expressing the FoxP3 transcription factor play a crucial role in the protection against undesired T cell activation and autoimmune disease, while still allowing a fast and effective immunological response to pathogens. To exert its role, FoxP3 interacts with an astonishing array of transcriptional regulators [7]. The complexity of this network makes predicting the effect of any mutation in the FoxP3 protein a daunting task. In this study, we found that the number of nTreg cells generated in the thymus of Tg4 FoxP3^gfp^ mice was not altered significantly, but lower numbers of FoxP3^+^ Treg cells in peripheral lymphoid organs of mature mice first suggested that iTreg cell development was impaired as a result of the N-terminal modification of FoxP3. This impaired iTreg cell differentiation was confirmed in vitro, but interestingly, in female Tg4 FoxP3^wt/gfp^ mice, we found that the mean frequency of GFP^pos^ cells among Treg cells in the thymus and periphery was not only often higher than the 50% expected from random X chromosome inactivation, it was also much higher than previously demonstrated in FoxP3-reporter K/BxN, NOD and C57BL/6 mice [5]. This could suggest that the nature of the MBP Ac1-9 peptide, for which T cells in the Tg4 model express a specific TCR, or other strain-specific characteristics somehow give GFP^pos^ cells a selective advantage during thymic generation. However, this does not explain the observation of the very strong correlation in GFP expression between thymic nTreg cells and iTreg cells generated in vitro from splenic naive CD4^+^ T cells of the same Tg4 FoxP3^wt/gfp^ female. The correlation would suggest that the skewed frequency at which the GFP-carrying X chromosome is active is determined prior to FoxP3 induction and maintained during both thymic and peripheral Treg cell development. In fact, X chromosome inactivation in mice may not be random but rather be partially imprinted [19]. This would imply that the maternal X chromosome has a slight advantage over the paternal one, thus skewing the frequency of active X-chromosomal genes in heterozygous animals. In our Tg4 FoxP3^wt/gfp^ females, where the GFP-encoding X chromosome was generally maternal, this would mean that the results were geared towards a higher frequency of GFP^pos^ Treg cells, as found. In theory, the difference between our results and those of others may, therefore, not reflect a difference in Treg development between the models but rather a difference in breeding strategy to obtain heterozygous females.

Based on their own findings, Darce *et al* suggested that the modification of FoxP3 leads to a stronger suppression of Th2 and Th17 cell-mediated arthritis, whereas protection against Th1 cell-linked diabetes is negatively affected due to the enhanced interaction of IRF4 with mutated FoxP3 [5]. In the Tg4 mouse, encephalomyelitis is initiated by Th1 cells but may also have elements of Th17 cell responses, although the latter are not strictly required for pathology [10]. It was, therefore, interesting to find that Tg4 FoxP3^gfp^ mice did not develop spontaneous EAE, did not show augmented peripheral lymphocyte activation or hyperproliferation even at 24 weeks of age, and showed no greater susceptibility to induced EAE. Moreover, the phenotype of GFP^pos^ nTreg cells suggested that they may have augmented suppressive abilities compared to GFP^neg^ nTreg cells in Tg4 FoxP3^wt/gfp^ mice, based on increased expression of common Treg cell markers such as CD103 and PD-1. This, surprisingly, phenotypically links the Tg4 model of EAE more to the K/BxN model of Th2/Th17-mediated arthritis than the NOD model of Th1-mediated diabetes.

The lack of increase in Foxp3^+^ Treg cell number in the spleens of aged Tg4 FoxP3^gfp^ males compared to younger equivalents suggested that peripheral induction of FoxP3 expression may be impaired. Indeed, both the use of peptide and antibody stimulation to generate iTreg cells, in vitro, demonstrated that the frequency of Foxp3 induction was negatively affected by the transgene. This was particularly apparent at peptide concentrations that yielded a lower conversion rate of Tconv cells to iTreg cells. Importantly, GFP^pos^ iTreg cells also showed a lower level of FoxP3 expression per cell, which may compromise their stability, as FoxP3 stabilizes its own transcription [20]. This was reflected in the stability of Tg4 FoxP3^gfp^ iTreg cells in co-culture with Tconv cells but not to the same level in conditions using Tg4 FoxP3^wt/gfp^ iTreg cells. In the latter case, the GFP^neg^ iTreg cells may have aided the stability of GFP^pos^ iTreg cells.

Overall, we demonstrate here that the N-terminal modification of FoxP3 impairs the generation of peripheral iTreg cells but not thymic nTreg cells in the Tg4 model. The latter seem equally or more functional than their wild-type counterparts and successfully protect against autoimmune disease. In the Tg4 FoxP3^gfp^ mouse, T cells express a TCR specific for a thymically expressed protein, which may explain why tolerance can be maintained by functional nTreg cells, even when peripheral conversion of autoreactive T cells to a suppressor phenotype is clearly impaired. Finally, these results indicate that the complexity of interactions of FoxP3 with other transcriptional regulators is such that seemingly unobtrusive mutations or polymorphisms may have dramatic and unpredictable effects, depending on the context.
